# Adolescent-Parent Dyad and Adolescent Eating Disorders: A Standardized Patient Simulation

**DOI:** 10.7759/cureus.101237

**Published:** 2026-01-10

**Authors:** Connie Gao, Carolyne Plansky, Nicole Ferguson, Jennifer Caputo-Seidler, Amy Weiss, Vinita Kiluk

**Affiliations:** 1 Medicine, University of South Florida Morsani College of Medicine, Tampa, USA; 2 Pediatrics, Connecticut Children's Medical Center, Hartford, USA; 3 Internal Medicine, University of South Florida Morsani College of Medicine, Tampa, USA; 4 Pediatrics, University of South Florida Morsani College of Medicine, Tampa, USA

**Keywords:** adolescent health, adolescent-parent dyad, eating disorders, medical students, observed structured clinical examination

## Abstract

Introduction: Many medical students are uncomfortable with adolescent patients and the associated difficulties of navigating speaking with an adolescent alone while ensuring confidentiality. To address this, students are encouraged to effectively use the home, education and employment, eating, activities, drugs, sexuality, suicide/depression, and safety (HEEADSSS) assessment, which is a history-taking method designed to reveal information about a patient’s home life, social life, and self-image. We created this observed structured clinical examination (OSCE) for learners to practice their communication skills and improve their comfort in seeing adolescents. In this case, learners interviewed an adolescent female with an overbearing mother, which adds yet another level of difficulty. Medical students practised history and physical exam skills as well as developed a differential diagnosis related to the chief complaint of the OSCE.

Methods: All 118 third-year medical students at a large academic medical institution in 2023-2024 participated in this OSCE. The OSCE was designed as a 25-minute interview of an adolescent and mother standardized patient (SP) pair. The learners were instructed to identify three differentials whilst navigating a tense adolescent-parent dyad. Students were evaluated by their faculty preceptor and SP. Acceptable differentials identified by faculty were quantified (mean and standard deviation) to assess student success in the OSCE. SP feedback was reported to gauge overall student performance.

Results: The response to this OSCE has been very positive. On average, students identified 2.24 correct differentials with a standard deviation of 0.71 differentials. On feedback forms, standardized patients (SPs) indicated that students met or exceeded expectations in 92% of encounters for partnership, 86% for empathy, 84% for apology, 93% for respect, 92% for legitimization, and 83% for support. While students admit the OSCE can be challenging, they also recognize the realism in the case and appreciate the opportunity to practice their skills. Students were able to take focused histories, perform focused physical exams, and create a list of relevant differential diagnoses. They also learned through experience and preceptor feedback about the importance of speaking privately to adolescents, maintaining confidentiality, and using the HEEADSSS assessment.

Discussion: This OSCE has provided an opportunity to increase medical students’ exposure to difficult adolescent scenarios and their ability to identify a wide breadth of potential diagnoses. It also encouraged students to build their skills in rapport-building and navigating challenging patient-family dynamics. Implementing direct feedback from SPs, either as a debrief or included in the preceptor rubric, would give students more access to adopt constructive feedback. The simulation could be similarly implemented in the training of interns and junior residents from pediatrics, internal medicine-pediatrics, family medicine, or psychiatry as well.

## Introduction

Medical students may find encounters with adolescent patients to be difficult for a variety of reasons. Balancing speaking privately with a teenager while maintaining confidentiality can be challenging, and questions regarding risk-taking behaviors, such as substance use or sexual activity, may be embarrassing for learners to ask. However, due to high rates of morbidity and mortality from risk-taking behaviors and unintentional injuries in adolescents, a comprehensive psychosocial interview is essential. Adolescents who report receiving patient-centered care are more likely to have participated in private conversations with a clinician as well as discussions regarding health behaviors. These same adolescents are less likely to report an unmet need for care or having a serious, untreated health problem [1]. 

The observed structured clinical examination (OSCE) presented here was created to primarily increase learners’ exposure to challenging adolescent patients and their parents, and to improve comfort level in having difficult conversations with this patient population. Recent studies have shown that medical students are reporting feeling underprepared for their pediatrics clerkships, therefore making OSCEs, such as the one presented in this paper, are more relevant than ever [2]. As pediatric clerkships have decreased in length over the past decade, there is no guarantee that students will develop the skills necessary to approach an overbearing parent and their ambivalent teenager [3]. Adolescent SPs have been shown to improve students’ skills of working with adolescent patients and to help students bridge their own past experiences of adolescence with their new role in a physician-patient interaction. Each additional practice with adolescent patients helps students fine-tune their professionalism in this complicated setting [4]. The OSCE format was chosen to decrease didactic time and increase student experiential learning. 

Understanding how to properly utilize the home, education and employment, eating, activities, drugs, sexuality, suicide/depression, and safety (HEEADSSS) assessment is an important skill for a physician to have, as is recommended by the American Academy of Pediatrics [5]. HEEADSSS is designed as a thorough yet succinct history, taking method that elucidates information about a patient’s home life, social life, and self-image [6]. Students were taught to use HEEADSSS as a tool to structure their interview given the discomfort many students have with adolescent patient interviews. It is designed to gradually increase the invasiveness of each question asked to avoid overwhelming the adolescent patient. This helps the physician develop rapport with both the patient and the patient’s parent over the course of the interview [6]. Learners have also been taught about the need to speak with adolescent patients privately, the need for and importance of confidentiality, and the limits of confidentiality. 

Furthermore, this OSCE focuses on developing diagnostic reasoning skills and obtaining a thorough history. It is particularly important that medical trainees gain experience in guiding conversations regarding adolescent mental health and eating behaviors. Physicians in primary care are in the position to detect eating disorders early and prevent worsening complications of the disease [7]. There has been a dramatic increase in the incidence of eating disorders following the coronavirus disease 2019 pandemic, primarily in female adolescents with diagnoses of anorexia nervosa. With such a staggering rise in hospital admissions for self-harm and eating disorders, early detection by physicians is more important than ever [8,9]. This case highlights several red flags, as well as more subtle signs, that can guide clinicians to consider further work-up for an eating disorder. At the same time, the patient presentation is purposefully ambiguous enough that students are required to develop a broad differential for amenorrhea in adolescents. Not only is it important for students to correctly identify the root of a patient’s problem, but it is equally important for learners to keep an open mind and avoid confirmation bias when conducting the rest of their history and exam. In this OSCE, students were asked to provide three potential differential diagnoses for the patient’s presentation, rather than focusing on one correct differential diagnosis. This serves as a measurable goal and encourages students to consider less readily obvious causes of amenorrhea and expand their diagnostic skills. 

## Materials and methods

Educational context 

This OSCE was first implemented in 2012. The simulation was created to ensure standardization and to guarantee that all students were exposed to a difficult adolescent patient scenario. We chose to use the OSCE format as an experiential form of learning for the students, which allows educators to assess students’ higher-level skills in communication with adolescent patients and management of this difficult clinical scenario. This format also allows the student to demonstrate the application and analysis levels of Bloom’s Taxonomy while entering into the beginning stages of evaluation for the students who were able to successfully discuss a management plan with this patient and mother. 

Setting and participants 

The simulation is generally run four times per year, with about one-fourth of the third-year medical school class participating each time. All third-year medical students at a large academic institution participated. Each time we run this OSCE, we use four rooms concurrently in our medical education simulation center, which are designed to imitate a clinic setting. Prior to the OSCE, students were educated on the HEEADSSS framework and were encouraged to follow its structure (Appendix A). Each adolescent SP has a large water bottle or Styrofoam cup in the room. No other props are needed. 

SPs selected for the adolescent patient in this case are typically in their early 20s but dressed to appear like high school students. SPs are asked to wear athletic clothing with baggy clothes overlaying (sweatshirt). We ask the SP mothers to dress well and appear like a middle-aged mother from an upper-middle-class family. Standardized patients and preceptors are emailed the standardized patient script (Appendix B) and the key teaching points (Appendix C) the week prior to the OSCE in order to review the material. All of our SPs receive general training on communication skills and providing feedback prior to working at our simulation center. On the day of the OSCE, the SPs receive training on this specific case. For example, the SPs playing the mother are asked to push back against the students’ request for them to leave the examination room unless rapport is achieved or if their questions from the learner are answered in an understandable manner. 

Case procedure 

At the start of the OSCE, students are given a door note (Appendix D) stating the chief complaint of amenorrhea and a set of vital signs. The door note includes instructions for the student to take on the role of the primary care doctor seeing the patient. The student is assigned to take a history related to the patient’s chief complaint. The student is asked to provide their top three differential diagnoses before leaving the room, along with an initial treatment plan. Students are given 25 minutes to conduct their patient interview and receive a five-minute warning prior to the end of the interview. 

Learner assessment 

After the OSCE, faculty preceptors and SPs are then asked to fill out written feedback forms (Appendix E) [10]. Students are asked to write-up the case with inclusion of patient history, assessment consisting of three differential diagnoses, and management plan. Students are asked to allow themselves 10 minutes for a write-up, timing themselves, and then must electronically submit the write-up within one week of the OSCE. These write-ups are reviewed by faculty or fourth-year medical students with formative feedback provided electronically. At the end of the year, students rated the case based on how they felt it helped advance their abilities to communicate with patients. 

Data sources and measures 

Analysis included all third-year medical students’ write-ups and SP data from the 2023-2024 academic year. The sample size was determined by the number of third-year medical students in the cohort during the 2023-2024 academic year; all eligible students were included. 

Faculty defined a priori the set of acceptable differential diagnoses and alternatives (Table 1). Student write-ups were screened, and the presence of acceptable differentials was recorded for analysis. A maximum of one eating disorder and one thyroid disorder diagnosis was accepted. Differential diagnoses of malnutrition or insufficient caloric intake were considered invalid if the student failed to provide an identified cause. Other differential diagnoses were excluded due to a lack of consistency with the patient’s history and exam. These include child neglect, depression, ovarian cancer, avoidant restrictive food intake disorder, primary anovulation, hypomania, anemia, human immunodeficiency virus (HIV), adrenal insufficiency, and polycystic ovarian syndrome. Quantitative analysis was descriptive. To gauge students’ understanding of the OSCE, the average number and standard deviation of acceptable differentials were obtained. Students’ end-of-year evaluation responses for the OSCE were reported. 

**Table 1:** Acceptable differentials and alternatives.

Differential	Acceptable Alternative	Not Accepted
Anorexia nervosa	Bulimia, Other not specified eating disorder	Avoidant/restrictive food intake disorder (ARFID)
Relative energy deficiency in sport (RED-S)	Athletic triad, female athlete triad, stress-induced amenorrhea, functional hypothalamic amenorrhea	Hormonal imbalance, hypogonadotropic hypogonadism, hypopituitarism, and adrenal insufficiency
Malnutrition secondary to Inflammatory Bowel Disease (IBD)	Malnutrition secondary to (other medical condition)	Malnutrition secondary to poor caloric intake (student must give medical cause), Iron-deficient anemia, Vague "nutritional deficiency" or "insufficient caloric intake"
Obsessive-compulsive personality disorder	Obsessive-compulsive disorder (OCD)	Depression, adjustment disorder
Pregnancy	-	-
Premature ovarian failure	-	Polycystic ovary syndrome (PCOS), primary amenorrhea, anovulatory cycles
Diabetes mellitus	-	-
Thyroidopathy	Hyperthyroidism, hypothyroidism	-
Body dysmorphic disorder	-	-
Hyperprolactinemia	Prolactinoma, pituitary adenoma	-

SP feedback responses were summarized and used to gauge overall student feedback. SPs rated each student on six domains: partnership, empathy, apology, respect, legitimization, and time management. We summarized the class performance, reporting the proportion of medical students who met or exceeded the SP’s expectation. SP short-answer responses were categorized into common themes (broadly positive or negative, then further into students’ ability to create comfort or a judgmental environment). The frequency of themes was quantified.

## Results

Since this OSCE was first implemented in 2012, about 1,560 third-year medical students have completed this OSCE at our institution. During the 2023-2024 academic year, 118 third-year medical students participated in the case and submitted write-ups available for our review. All third-year medical students' write-ups were included in our analysis. In examining the differential diagnosis of 118 write-ups, one (0.8%) write-up included 0 valid differential diagnoses, 16 (13.6%) write-ups included only one valid differential diagnosis, 55 (46.6%) write-ups included two valid differential diagnoses, and 46 (39.0%) write-ups included three valid differential diagnoses. 

Students are instructed to include three differential diagnoses within their case write-ups, and 110 (93.2%) of 118 submissions met this goal. However, of the 340 differentials given, only 264 (77.6%) met the study's a priori criteria for a valid differential. On average, students identified 2.24 correct differentials with a standard deviation of 0.71 differentials. As shown in Table 1, we excluded differential diagnoses that were redundant, nonspecific, or inappropriate for this patient scenario. 

**Figure 1:**
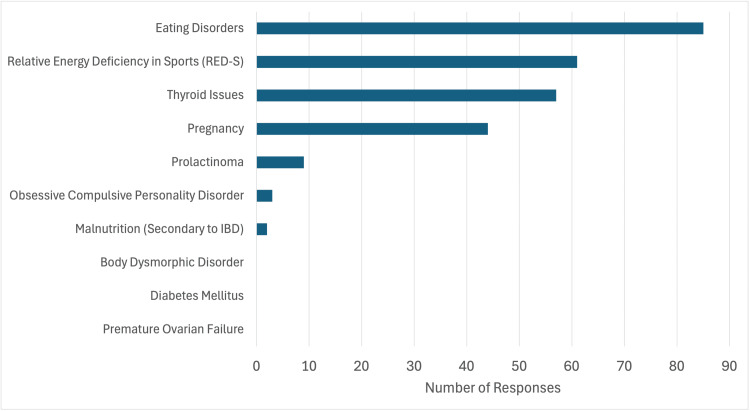
Number of students that identified particular diagnoses. Compares acceptable differentials and the number of times students proposed the differential after their interaction with the SPs.

In examining the 264 valid differential diagnoses (shown graphically in Figure 1): 85 (72.0%) students identified eating disorders, 61 (51.6%) students identified relative energy deficiency in sports, 57 (48.3%) students identified thyroid disease, 44 (37.3%) students identified pregnancy, 9 (7.6%) students identified prolactinoma, 3 (2.5%) student identified obsessive compulsive personality disorder, 2 (1.7%) students identified malnutrition secondary to inflammatory bowel disease, 0 (0.0%) students identified body dysmorphic disorder, and 0 (0.0%) student identified premature ovarian failure. 

The remainder of our educational objectives cannot be objectively measured but instead are discerned from feedback forms from the SP’s and students. On feedback forms, SPs rated students on their abilities to project partnership, empathy, apology, respect, legitimization, and support during the OSCE (Appendix E) [10]. Students met or were above expectations 92% (N=108) of the time in partnership, 86% (N=101) of the time for empathy, 84% (N=99) of the time for apology, 93% (N=109) of the time for respect, 92% (N=108) of the time for legitimization, and 83% (N=97)of the time for support (Table 2). 

**Table 2:** SP feedback responses when asked about student traits during OSCE.

Score	Partnership	Empathy	Apology	Respect	Legitimization	Support
Above expectations	50%	47%	39%	49%	51%	52%
Meets expectations	42%	39%	24%	45%	41%	31%

The SPs then gave feedback detailing how students made them feel, how they could improve next time, and what the students did well (Appendix E). Themes in the SPs' feedback were identified and shown below (Table 3).

**Table 3:** Standardized Patient (SP) feedback response to how the student made them feel.

Standardized Patient Feedback Prompt: “As a patient, these students made me feel”		
Positive themes	Number of Students	Example Statement
Comfortability	70 (59.3%)	"As a patient, the student made me feel very comforted and heard"
Listened to	32 (27.1%)	"I felt partnered with the student to figure out what is going on. "
Cared for	21 (17.8%)	"As patients, “J” made us feel important. He was incredibly warm and engaging."
Feeling partnered/included in conversation	17 (14.4%)	"I felt truly respected, supported, and partnered at the highest level."
Negative themes	Number of students	Example Statement
Lack of rapport	5 (4.2%)	"The student made us feel uncomfortable since he didn't establish rapport before asking awkward questions."
Overwhelmed	3 (2.5%)	"As a patient, the student made me feel like he overwhelmed us with a lot of information."
Judged	3 (2.5%)	"As a patient, this student made me feel overall supported but a little judged."

SPs noted that students made them feel comfortable, listened to, cared for, and partnered with. In the feedback, the most prominent themes critiquing the students involved a lack of rapport before asking sensitive questions, feeling overwhelmed, or feeling judged by the students. When asked for suggested improvements for students (Table 4), SPs noted building rapport, navigating timing when asking the mother to leave, decreasing medical jargon, and building confidence.

**Table 4:** Standardized Patient (SP) feedback response to how the student could improve.

Standardize Patient Feedback Prompt: “Please state what the students could improve on for next time.”	
Response from SP	Number of students receiving feedback
Building rapport with patient before asking sensitive questions	10 (8.5%)
Navigating timing and how to ask the mother to leave	10 (8.5%)
Decreasing medical jargon use	5 (4.2%)
Confidence/knowing next steps	4 (4.2%)

When asking for feedback on what the student did well (Table 5), SPs stated the students frequently made them feel comfortable, had clear communication, made them feel heard, and would ask the mom to leave well. 

**Table 5:** SP feedback response to what the student did well.

Standardize Patient Feedback Prompt: “Please state what the students did well.”	
Response from SP	Number of students recieving feedback
Making the patient feel comfortable	20 (16.9%)
Clear communication/clear explanations	20 (16.9%)
Feeling heard/listened to	15 (12.7%)
Asked the mom to leave well	10 (8.5%)

Additionally, preceptors reviewed what eating disorder red flags the students picked up in their interviews. SPs and preceptors discussed the students’ ability to successfully ask the mother to leave the room and discuss confidentiality, with a focus on the students’ ability to develop a rapport with the adolescent and earn the mother’s trust. Often, students who were not able to build a relationship with the SPs were not successful in getting the mother to leave the room. Preceptors also assessed the student’s plan for patient follow-up and evaluated the student’s use of the HEEADSSS assessment. 

Several faculty members have acted as preceptors for this OSCE at our institution over the last few years. Typical faculty preceptors used for this case specialized in pediatrics, adolescent medicine, internal medicine-paediatrics, or family medicine. Faculty comments about the case are generally positive. Our faculty have enjoyed precepting this case, citing the realistic nature of the scenario and the opportunity for student education and growth. Concerns cited by faculty include the short amount of time given to the students and the fact that many students become fixated on getting mom out of the room, thereby wasting valuable interview time. 

A cadre of younger women SPs (for the adolescent patient) and older women SPs (for the mom) have participated in this OSCE over the past few years. Many SPs comment that it is one of their favourite cases to play. 

**Figure 2:**
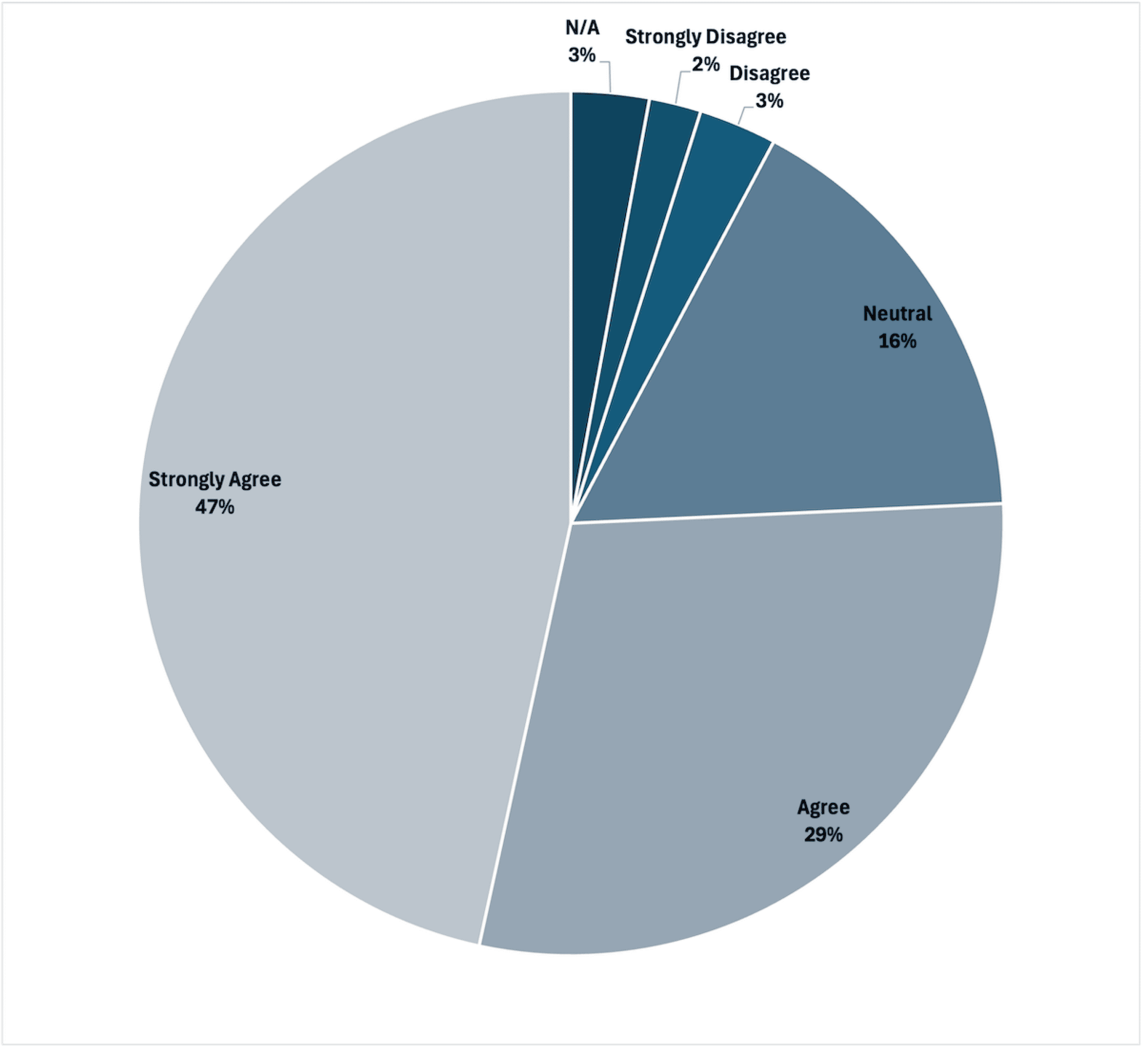
Student response to "The adolescent suicide case and eating disorders cases improved my comfort level and ability to communicate with adolescent patients and their parents."

At the end of the year, students were given a course feedback form that gauged their feedback regarding the doctoring class. Particularly, 100 students responded to the prompt “the adolescent suicide case and eating disorder cases improved my comfort level and ability to communicate with adolescent patients and their parents." Of the 100 students responding (shown in Figure 2), 47 strongly agreed, 29 agreed, 16 were neutral, 3 disagreed, 2 strongly disagreed, and 3 stated not applicable. 

## Discussion

We have found that this OSCE has provided an opportunity to increase exposure to adolescent patients, specifically a difficult adolescent scenario with an overbearing parent. There are many ways in which this case allows students to hone their communication skills. The learner must navigate seeking information from a reticent adolescent when her overbearing mom keeps answering questions. Many students struggle with the issue of asking their mom to leave the room to speak to the adolescent patient privately. Some students become fixated on getting mom out of the room to the detriment of their overall performance. They forget about the broader goals of rapport building and information gathering as they instead spend time arguing with mom. Some students are blatantly rude to the mother, while others discount her. Other students ignore the adolescent patient or aren’t able to build rapport with her since the mom is so overbearing. 

The amount of time given for the interview is also a challenge for students. Many students would benefit from more time to talk to the adolescent and build rapport. However, due to the desire to keep the case realistic as would occur in a time-crunched clinic and also time limitations within our simulation center, we have decided to keep the time limited to 25 minutes [11, 12]. The students were given the HEEADSSS assessment to help mitigate the time constraints and provide structure for forming rapport with both the parent and adolescent. The HEEADSSS report provided students with an organized approach to efficiently gather social information, minimizing stress and maximizing the information they collected. 

Despite the challenge, most students appreciated the opportunity to participate in this OSCE, with 76% (N=76) of the students agreeing or strongly agreeing that the OSCE helped them gain skills when communicating with adolescent patients and their family members on their end-of-year feedback forms. However, the students' responses to both an adolescent suicide OSCE and the eating disorder OSCE discussed in this paper were gauged in a single prompt in the feedback form; therefore, it is difficult to elucidate how the students felt about this OSCE in particular. The overall responses to the OSCE were still favorable, indicating the students valued their experiences, with nearly half of the class stating they strongly agreed with the prompt. 

Additionally, one of the key goals of the case was to ensure students could generate a comprehensive diagnostic differential. Early in clinical practice, students fail to expand their differential, especially when patients with particularly salient symptoms like amenorrhea present [13,14]. On average, students identified more than one acceptable differential (2.24 acceptable differentials on average). This indicates that despite the challenging nature of the interpersonal relationships of the OSCE, students were able to synthesize the information to reliably consider alternative differential diagnoses such as anorexia nervosa, relative energy deficiency in sports, thyroidopathy, etc. 

SP feedback was used to gauge the students’ abilities to create rapport and manage the adolescent-parent dyad. Overall, the SPs stated that students were able to respond to the case with empathy, respect, and support while creating partnerships with them. Most frequently, SPs positively commented on students’ abilities to make them feel comfortable and on students' abilities to give clear explanations. However, SPs noted that students could still work on timing and building rapport before asking sensitive questions. Overall, the feedback indicated that students were able to gain practice with managing the adolescent-parent dyad and helped identify key areas where students could continue to grow.

A limitation of this OSCE is that students received feedback from the SPs through an online service, but given difficulties with granting permissions, many students did not check their feedback. As a key objective of the case is preparing students as clinicians, receiving SP feedback should be prioritized as it can help students adjust their interpersonal navigation practices in preparation for future patients. To address this, SPs could leave feedback in the same rubric that the preceptors fill out; therefore, the preceptors' and SPs’ feedback are stored in one place and are easily accessible for the students. Students could also be given designated debrief or feedback time with the SP and preceptors [15]. Additionally, as the OSCE was integrated into an existing longitudinal medical student course and needed to be conducted in a timely manner, the students were not required to conduct pre and post-comparisons on performance, nor was a control group implemented. To strengthen the study's results, both of these interventions could be implemented to obtain a more quantitative evaluation. 

This OSCE was successful in helping medical students gain practice when working with complex adolescent health dynamics. Students gained insight into navigating parent-child dyads and creating valid differential diagnoses despite challenging circumstances. Given the positive feedback from students, SPs, and faculty and ease of setup, the case could be used in a variety of environments to help medical students at other programs, interns, and even residents. 

## Conclusions

Overall, the OSCE met the objectives set out initially and was seen favorably by SPs, students, and faculty members. The OSCE gave students practice in improving the comfort levels of adolescents and navigating family dynamics as they interact with healthcare professionals. The OSCE can be used to improve students’ abilities to navigate adolescent health cases and family dynamics while being low-cost and effective. 

## Appendices

Appendix A

HEEADSSS interviewing tool. Adapted from Klein DA et al  (Table 6) [6].  

**Table 6:** HEEADSSS assessment.

Adolescent History	Potential First-Line Questions
Home	Who lives with you? Where do you live? What are relationships like at home? Can you talk to anyone at home about stress? Is there anyone new at home?Has someone left recently?
Education and employment	Tell me about school. Is your school a safe place? Have you been bullied at school? Do you feel like you belong at school? Are there adults at school you feel you can talk to about something important? Do you have any failing grades? What are your future education or employment goals? Are you working? Where? How much?
Eating	Does your weight or body shape cause you any stress? Have there been any recent changes in your weight? Have you dieted in the last year? How? How often?
Activities	What do you do for fun? How do you spend time with friends or family? What types of things do you use the Internet for? How many hours per day do you spend in front of a screen (TV, computer, phone)? Do you wish you spent less time on these things?
Drugs	Do any of your friends or family members use tobacco? Alcohol? Other drugs? Do you use tobacco, or electronic cigarettes? Alcohol? Other drugs, energy drinks, steroids, or medications not prescribed to you?
Sexuality	Have you ever been in a romantic relationship? Tell me about the people you’ve dated. Have any of your relationships been sexual relationships (such as involving kissing or touching)? Are you attracted to anyone now? Are you interested in boys? Girls? Both? Not yet sure?
Suicide/depression	Do you feel more stressed or anxious than usual? Do you feel sad or down more than usual? Are you “bored” much of the time? Are you having trouble going to sleep? Have you thought a lot about hurting yourself or someone else?
Safety	Have you ever been seriously injured? Do you always wear a seatbelt in the car? Have you ever met in person with someone you first encountered online? When was the last time you sent a text message while driving? Tell me about a time you have ridden with a driver who was drunk or high. When? How often? Is there a lot of violence at your home or school? In your neighborhood? Among your friends?

Appendix B

Standardized patient script (Table 7).

**Table 7:** Standardized patient script.

Case Information: SPs should not volunteer information unless specifically requested. Responses should be given in the patient’s own words. Cassidy is polite, but she does not volunteer any information. She should have a big gulp cup with her, which she drinks out of frequently during interview-it is filled with water. Mother is overbearing, and given the chance, she will answer all the questions for Cassidy.	
Setting	Clinic, new patient visit
Props	Cassidy should have a big gulp filled with water that she is drinking from periodically during the interview.
Opening statement	Mother: “She has not had her period for a couple of months, and I wanted to get her checked out.”
History of present illness	
Onset	Mother: “For a couple of months”
Severity	Mother: “No spotting. No cramping. She all of a sudden stopped getting her period.”
What makes it better	Mother: “Not sure”
What makes it worse	Mother: “Not sure”
Associated symptoms	Mother: “She started her period in fifth grade-she was one of the first in her class, about one year earlier than her friends. She did not know what was going on, she had not seen the video in PE class yet” (mother will speak like it is kind of funny). Cassidy should show on her face that this was an uncomfortable event for her. Mother: “She got it monthly since then, and it was about five days long. She never really had any cramps or heavy bleeding. She went through five pads per day, which were not soaked through.”
Review of systems: All negative with the exception of lightheadedness and hair thinning	
Onset	Cassidy: “I noticed hair loss over the last couple of weeks. The lightheadedness started about one month ago.”
What makes it better	Cassidy: “Not sure how to make the hair loss better. I’ve been trying new shampoos, but they have not helped. If I don’t get up too quickly, I won’t feel bad light-headed.”
Past medical history	
Per Cassidy	“I haven’t been sick for a long time. Especially this year, most of my friends have had sore throats-they missed a bunch of days of school, and I just did not get sick- I guess I have been really healthy."
Per mother	Her history is unchanged: no asthma, no new diagnoses, shots up to date.
Medications	Cassidy does not take any medications
Allergies	No known allergies to medications or environment.
Family history	
Per mother	“I have OCD, which is treated and fine now. Paternal uncle is an alcoholic. Her father was just diagnosed with angina (chest pain) and has a history of slightly elevated cholesterol. Her younger sister is healthy.”
HEEADSSS assessment	
The student should ask their mother to leave the room for this part of the interview. They should discuss that this is the part of the interview where it is important to talk with Cassidy on her own to foster the doctor-patient relationship.	Mother does not want to leave the room. She states that she knows her daughter very well. Mother can also say that she knows the answers to everything the student is going to ask already, and they don’t have any secrets or keep anything from each other. Mother can say that Cassidy would want her to stay. Initially, mom should be adamant about not leaving the room, but can waive or change your mind depending on comfort level. Directions to SP mother: You can decide whether or not to leave the room based on what the student says and how comfortable you would be as a mother leaving your daughter in the room with a medical student without you. If the student makes you feel comfortable, then it’s ok to leave the room. However, if you are at all uncomfortable with how the student acts or asks you to leave, then don’t leave the room. In your feedback to the student at the end, you can let them know what they did/said that allowed you to leave the room or made you not want to leave. Directions to SP Cassidy: You can decide to ask mom to stay if you do not feel comfortable, or tell mom it’s ok to leave you alone with the student if you are feeling comfortable. You can give feedback to the student about what swayed your decision at the end of the case. Things that might make you feel more comfortable with the student include (but aren’t limited to): the reason given for wanting to talk to Cassidy by herself, how respectful the student is, and the language and body language of the student.
Home: Who lives at home with Cassidy?	Mother: “Me and her father and her sister”
Exercise	Cassidy: “I have to run a lot for cross-country"
Activities/hobbies	Cassidy: “I am in a few clubs.”Mother: “I think I have noticed that you have been home more than you used to be-you have not been at Spanish club or the Yearbook like you used to last year. She has been studying more and more. Her straight A’s have become A+s and she is on a special honor roll currently. I am so proud of her.”; Cassidy: “I still do cross-country.”Mother: Yeah, a few weekends ago, I thought she was talking on the phone with one of her friends, but she was doing extra practice for cross country. I thought it was weird at the time.”; Cassidy: “I just like running.”
Diet	Mother: “We eat a healthy American diet.”; If asked more about the diet... Cassidy: “I went to an overnight camp about healthy living for a few weeks with some of my friends, and we thought it would be fun to start eating really healthy while we were there. They gave us a lecture on counting calories, and I thought this was fascinating. I realised at home we were not eating all that healthy, and I have made some suggestions, and I think we are eating a whole lot healthier now.”Mother: “You are right, honey, we are eating a lot better now, and I am glad, since your dad has that high cholesterol.”; When asked specifically how much she eats on a daily basis, Cassidy said, “I am eating more than enough.”; If asked specifically what her daily calorie intake is... Cassidy: “It is about 1200-1500 calories a day.”If Cassidy is pressed further with something like “Would you tell me what you had to eat yesterday, beginning when you woke up in the morning and ending when you went to sleep for the night?”... Cassidy: “For breakfast, I had milk. For lunch, I had water and fruit with cottage cheese, and for dinner I had some salad, chicken, and some water.”; If asked specifically what milk she drinks... Cassidy: “Skim”; If how much... Cassidy: “A cup”; For lunch and dinner, if asked further how much... Cassidy: “The skim milk, cottage cheese, and a plate-sized portion for both lunch and dinner”; If she is asked to quantify how much water she drinks in a day... Cassidy: “5-6 cups”; If the student should ask the mom if she can verify that amount for her meals.... Mother: “Oh, yes, she is eating so healthy these days.”
Weight: Have you had any weight changes recently?	Cassidy: “Not much. I think I weigh about 115 lbs, so I think maybe about 3 lbs weight loss since my well visit 6 months ago”
Body image	Mother: “She has always been uncomfortable with how pretty she is.”; Cassidy: “I think if I lost a few more pounds, maybe…I am not sure…I don’t know…”
Tobacco/alcohol/drug use	Mother: “My daughter has goals for her life, she would never let these things get in the way-she would never do these things.”
Sex/relationships	Cassidy: “No way!”
Occupation	Mother should make it known that they are upper middle class, that dad is a powerful attorney who has a big case he is working on, so that’s why he could not make it today. Mother is a successful interior designer.
Safety/abuse	If asked about abuse... Cassidy: (hesitates and looks down at feet) “No.” If a student asks why you are looking down or picks up on your hesitation, say some boys have been commenting on her appearance and how cute she is, and it has made her really uncomfortable.
Education	Mother: “She makes straight As.” If asked what she wants to be when grown up... Cassidy: “I want to be a doctor.”
Depression/life stressors	Cassidy: “No school is easy for me. Track is easy for me.” Mother: (in a hushed voice) I think she is a little worried about her father-she does not want to let on, but he has had a little health scare. He has always had trouble with his weight and has had high cholesterol, and now he has started having some chest pain. His doctors don’t think it was a heart attack just a bit of a wake-up call that he needs to have less stress and eat better.”
Follow-up and management plan	
What if I told you your weight is 108 lbs today?	Cassidy: “That can’t be, I am pretty sure I weigh 115 lbs. That is a lot less than I thought I weighed.”
Your low heart rate, with light-headedness when you get up, the loss of your period and your thinning hair make me worry that you have lost more weight than your body would like, that you are below what we call an ideal body weight. Were you trying to lose weight?	Cassidy: “I don’t know. I was really just trying to have a healthy diet for my family and me. I thought I just lost 3 pounds”
Last time we saw you, your height was 5’4’’, and you weighed 118 lbs. That was pretty close to where your weight should be for your height.	Cassidy: “Are you sure? I was at an urgent care 3 months ago with one of my sick friends, and I weighed myself, and I was 115, and I did my height, and I was 5’4’’. When did this happen?”
I would like to follow you really closely to help you get to your ideal body weight, at the beginning every 2-3 days. I know a specialist who has a healthy weight clinic, and I would like you to see her. There, you will be able to see a nutritionist and a therapist that deals with these types of issues to help you. I want to see if we can get you in at the healthy weight clinic today, and we can get started. I think we can do this as an outpatient. How does that sound to you?	Mother: “Whatever it takes to help my daughter.” If Cassidy is asked for her thoughts... Cassidy: “I would not mind seeing a nutritionist. Maybe a therapist if you think I really need to see one.”

Appendix C

Key teaching points (Table 8).

**Table 8:** Key teaching points.

Learning Objective	Points of Emphasis
1. Identify anorexia (beginning of an eating disorder), and in particular identify anorexia (eating disorder) coupled with extreme exercise.	Cassidy has stopped all her other activities, but she is still running a lot. She is getting straight A+ but not talking to her friends on the phone as much. Cassidy is ambivalent regarding her weight; this is one of the signs that she may be fit for outpatient treatment.
2. Identify potential anorexia triggers.	Cassidy’s father has now been diagnosed with an illness, and she is worried. By restricting how much she eats, she feels (unconsciously or possibly consciously) this is a way she is gaining back control to compensate for a lack of control over her father’s health.
3. Understand the importance in adolescent medicine to have the parent leave the room to discuss personal and private issues with the adolescent.	In this case, the mother is very overbearing, and Cassidy is fine with that, but it is still important to have asked that mom leave the room and explain to the mother why. Mother may or may not leave in this case, but the student should attempt to speak to the adolescent alone. Please go over what should be discussed with the patient about confidentiality (meaning and exceptions to confidentiality such as suicidal or homicidal ideation).
4. Understand the importance in adolescent medicine to have close follow-up on the patient to ensure compliance with treatment plans since lack of compliance is possible.	In this particular case, we want to make sure we have close follow up to monitor Cassidy. It is important for students to recognize she is in a fragile situation and could easily shift toward requiring inpatient management.
5. To practice the H(home), E(education), E(eating), A(activities), D(diet), S(suicidality), S(sexuality), S(safety) assessment, which is a way to obtain pertinent history from the adolescent patient.	Please review with students if they have missed a piece of the assessment.

Appendix D

Door note for students (Table 9).

**Table 9:** Door note for students.

Scenario	
Chief complaint	“She missed her period the past couple of months.”
Setting	Outpatient clinic
Type of encounter	New patient
Vital signs	Blood pressure: 100/65; pulse: 55; temperature: 98.0 F; respiratory rate: 13; weight today: 108 lbs, body mass index (BMI): 18; weight 6 months ago: 118 lbs, BMI: 20
Nurse’s notes	Cassidy Brown is a 16-year-old female who has missed several periods and is in the clinic with mom today to determine what is going on.
Student’s role	You are the primary care doctor seeing the patient. Please list your 3 top differential diagnoses before you leave the room (along with the initial treatment plan).
Student assignment	25 minutes with patient: Take a history relative to the patient’s chief complaint and come up with an assessment/management plan to explain the patient’s symptoms. No physical exam is needed.
Student objectives	
1. Obtain a focused history in a patient with a chief complaint of missing several periods.	
2. Formulate the most likely diagnosis and a list of at least 3 other differential diagnoses.	
3. Consider the most appropriate initial diagnostic tests to order.	
4. Formulate a treatment plan and provide appropriate counselling and education to the patient. These may include: a. Inpatient versus outpatient treatment, b. Medications to be ordered, c. Procedures that may be needed, d. Referrals that may be needed, e. Follow-up that may be needed, f. Lifestyle modifications that may be needed, g. Patient education that may be needed.	

Appendix E

Standardized patient feedback form (Table 10) [10]. 

**Table 10:** Standardized patient feedback form.

Standardized Patient Feedback Form	
Multiple hoice	
1. Partnership: The student worked with you to identify the main concerns (i.e. “Let's deal with this together”, “We can do this...”, or use of similar phrases).	Options: Not done; below expectations: inconsistently – the student did not attempt to partner with you very often; meets expectations: mostly - the student often partnered with you and worked to identify your concerns; above expectations: consistently - the student consistently demonstrates a willingness to partner with you due to their shared interest in your health.
2. Empathy: The student acknowledged and demonstrated understanding of your feelings (i.e. “That sounds hard”, “You look upset”, or use of similar phrases).	Options: Not done; below expectations: inconsistently – the student did not acknowledge your feelings often; meets expectations: mostly - the student often acknowledged your feelings; above expectations: consistently - the student consistently acknowledged your feelings and verbalized this in empathy statements.
3. Apology: The student took personal responsibility where appropriate (i.e. “I'm sorry this happened to you”).	Options: Not done; below expectations: inconsistently - the student rarely took personal responsibility (where appropriate); meets expectations: mostly - the student frequently took personal responsibility (where appropriate); above expectations: consistently - the student consistently took personal responsibility (where appropriate); not applicable.
4. Respect: The student valued your choices, behaviors, and decisions, and was non-judgmental in their discussions with you.	Options: Not done; below expectations: inconsistently - the student did not always value your decisions; meets expectations: mostly - the student often valued your decisions and was non-judgmental in discussions with you; above expectations: consistently - the student consistently valued your decisions and was non-judgmental in discussions with you.
5. Legitimization: The student validates and shows understanding for your feelings and choices (i.e. “Anyone would be concerned with these symptoms").	Options: Not done; below expectations: inconsistently - the student rarely used words that validated and showed understanding of your feelings; meets expectations: mostly - the student frequently used words that validated and showed understanding of your feelings; above expectations: consistently - the student always used words that validated and showed understanding of your feelings.
6. Support: The student offered you support. (i.e. “I am here to help determine the cause of your symptoms”)	Options: Not done; below expectations: inconsistently - the student rarely used words that reflected their support of you as a patient; meets expectations: mostly - the student frequently used words that reflected their support of you as a patient; above expectations: consistently - the student always used words that reflected their support of you as a patient.
Open response	
7. “As a patient, these students made me feel”	
8. Please state what the student did well	
9. Please state what the student could improve on for next time	
